# Effects of a Lifestyle-Based Physical Activity Intervention on Medical Expenditure in Japanese Adults: A Community-Based Retrospective Study

**DOI:** 10.1155/2016/7530105

**Published:** 2016-07-14

**Authors:** Yasuyo Yoshizawa, Junghoon Kim, Shinya Kuno

**Affiliations:** ^1^Tsukuba Wellness Research Co., Ltd., Kashiwa, Chiba 277-8519, Japan; ^2^School of Nursing, Tokyo Women's Medical University, Tokyo 162-8666, Japan; ^3^Department of Sports Medicine, Graduate School of Comprehensive Human Sciences, University of Tsukuba, Tsukuba, Ibaraki 305-8577, Japan; ^4^Department of Preventive Medicine, Gachon University College of Medicine, Incheon 21999, Republic of Korea

## Abstract

*Background.* This study aimed to investigate whether a lifestyle-based physical activity program could contribute to reduced medical expenditure.* Methods.* The study participants were 60 adults aged 63.1 (standard deviation, 4.4) years in the intervention group; the case-control group consisted of 300 adults who were randomly selected from Japan's national health insurance system. This community-based retrospective study incorporated a 3-year follow-up.* Results.* The total and outpatient medical expenditure in the intervention group were significantly lower than in the control group: total expenditure, $US640.4/year; outpatient expenditure, $369.1/year. The odds ratio for outpatient visiting was 6.47-fold higher in the control than in the intervention group. *Conclusion.* Our study suggests that a health program to promote physical activity can result in reduced total medical expenditure, outpatient medical expenditure, and possibly also inpatient medical expenditure.

## 1. Introduction

It is predicted that the global population will age rapidly during the next half century [1]. The increase in medical expenditure associated with aging has become a public health problem worldwide [2]. Japan has an aging society, and it is expected to become an aged country (considered to be when the elderly population aged ≥ 65 years exceeds 39.9% of the total) in 2060 [1]. The increase in population aging in Japan is greater than in many other countries. In addition, lifestyle-related endocrine or metabolic diseases account for 41.1% of medical expenditure for primary disease in 2010; musculoskeletal conditions account for 7.6%. These two categories thus constitute 48.7% of costs for primary disease [2]. It is therefore extremely important for Japan to develop effective prevention of lifestyle-related diseases, extend healthy life expectancy, and thereby reduce the associated economic burden.

Japan has a national health insurance (NHI) system, and all residents are covered by some form of medical insurance. With NHI, major health services, such as health promotion, disease prevention, medical treatment, and rehabilitation, operate at a cost that is affordable by all people [3]. It has been determined that medical expenditure in Japan rose from $US38 billion to $1,907 billion during the 30-year period from 1960 to 1990 [4–6]. Moreover, the system that operates at deficit and developing measures to address this situation has become an issue of national priority [7].

According to a growing body of evidence, regular physical activity is significantly associated with decreased risk of noncommunicable diseases and mortality [8–12]. Several studies have reported that higher physical activity is negatively associated with medical expenditure [13–15]. For example, a population-based cohort study using a national dataset of Japanese adults found that medical expenditure was significantly reduced in individuals who spent longer time walking [13]. Similar results have been obtained in other countries [16–18]. Several studies have reported the effects of physical activity on medical expenditure, including Japan. However, most of those studies used an observational design [13, 14]. Accordingly, they could not exclude the possibility of reverse causation, whereby healthier individuals participated in physical activity and accordingly had lower medical expenditure. In addition, prior health problems may have had an effect on the lack of physical activity; previous physical health problems could also result in increased medical expenditure.

To the best of our knowledge, no intervention study has been reported about increasing physical activity to reduce medical expenditure. It is therefore necessary to develop and assess a community-level intervention program for promoting physical activity toward reducing healthcare costs. Thus, the objective of the present study was to investigate the effects of a lifestyle-based physical activity program, supported by a local government, on changes in medical expenditure compared with a randomly selected matching control group from a community over a 3-year observation period.

## 2. Materials and Methods

### 2.1. Study Participants

The participants in the intervention group were 60 individuals who engaged in a 9-month physical activity program in Kazo, Japan. Kazo is a city located in Saitama Prefecture. In 2011, it had an estimated population of 117,339, with 42,778 households, and a population density of 879.14/km^2^; the total area is 133.47 km^2^ [19]. The participants in the control group were randomly selected to match those in the intervention group from among 11,399 individuals who subscribed to NHI between January 2010 and December 2013. The participants in the intervention group were also enrolled in NHI. We selected control group participants using a case-control matching (CCM) method as a quasiexperimental design using SPSS (PASW Statistics 20 for Windows, IBM Inc., Armonk, NY, USA). The CCM method is able to accommodate matching for confounding variables to account for preexisting differences, thereby reducing selection bias and improving internal validity. In this study, we considered age, sex, and baseline medical expenditure as matching variables. To determine the effects of the physical activity intervention, we selected 300 participants for the control group. All the participants received a full explanation of the study purpose, and they provided their written informed consent to take part. The study protocol was approved by the Institutional Review Board of the Graduate School of Comprehensive Human Sciences, University of Tsukuba (numbers 24–27).

### 2.2. Measures of Physical Activity and Fitness

To measure their daily life physical activity, participants were required to wear a pedometer (HJ-730IT, Omron Healthcare, Kyoto, Japan) for 7 days before the start of the physical activity program. We measured total and brisk-walking step counts using the pedometer at baseline and every day during the intervention. We defined brisk walking as a pace equivalent to ≥4 metabolic equivalents and at least 10 min of continuous walking [20]. We also measured physical fitness using a test consisting of six items based on the Japan Fitness Test for physical activity programs: handgrip strength (kg); sit-ups (times/30 sec); sit-and-reach (cm); one-leg standing with open eyes (sec); walking speed (a 10 m hurdle walking test, sec/10 m); and aerobic capacity (watts/kg).

### 2.3. Anthropometric Assessment

We calculated the body mass index (BMI) as the body weight (kg)/height (m^2^). We measured the body fat mass index (percentage) and weight-adjusted skeletal muscle mass (percentage) using a bioelectrical impedance device (HBF-352, Omron Healthcare).

### 2.4. Physical Activity Program

All subjects in the intervention group participated in the lifestyle-based physical activity program to promote daily life activity. They performed aerobic exercises using a bicycle ergometer and doing weight-bearing exercises in classroom sessions twice a week. The subjects performed aerobic exercise for 30 min/day; the target intensity for the bicycle ergometer was calculated to be the physical work capacity that was 75% of the maximum heart rate [21]. The purpose of the weight-bearing exercise was to increase the mass of the psoas major muscles, and it consisted of seven items: squats, sit-ups, push-ups, leg extensions, leg curls, hip extensions, and back extensions (all were repeated 10 times in two sets) [21]. The participants were also instructed to increase their daily total step count to 3000 steps and to perform the weight-bearing exercise 3 days/week at home. With the step count, ≥2000 steps/day had to be conducted with brisk walking [20, 22]. We set the total physical activity levels in accordance with the recommended levels in current guidelines [23]. Every month, we gave the subjects feedback reports, summarizing their adherence to the target physical activity level. The intervention lasted for 9 months, from March 2011 to November 2011; we provided further support for the participants' self-management in a physical activity program over the subsequent 6 months.

### 2.5. Medical Expenditure

In Japan, all residents are covered by some form of medical insurance. NHI is one medical insurance system, and it covers about 35% of the population; the primary subscribers are the self-employed, agricultural workers, housewives, and retirees [3]. NHI covers almost all medical expenditures, such as diagnosis, examination, treatment, surgical operations, medical consumables, physical therapy, inpatient nursing care services, dental treatment, and other medical outlays [3]. We collected data related to medical cost claims from all participants who subscribed to NHI between January 2010 and December 2013 in Kazo [2, 19]. The medical claims data included the number of outpatient visits and inpatient care as well as the medical costs for outpatient and inpatient care. Those medical claims data were linked to the participants in the intervention group through the beneficiaries' ID numbers. For the baseline, we used medical expenditure data from January to December 2010. Based on previous studies, we also calculated medical expenditure per capita/year during the intervention and follow-up [14]. In this study, we converted monetary data to US dollars at the exchange rate of $1.00 that equals 108 yen.

### 2.6. Statistical Analysis

We expressed the participants' baseline characteristics as means and standard deviation (SD) and as percentages. We analyzed the mean difference in age and total, inpatient, and outpatient medical expenditure between the intervention and control groups using the Mann-Whitney *U* test. We used *χ* ^2^ test to evaluate the statistical significance among the categorical variables (frequency of males and females, elderly population, and obesity).

We determined whether the physical activity intervention contributed to change in medical expenditure: we examined the association between the physical activity program and average annual change in total, outpatient, and inpatient medical expenditure from baseline; we did so using mixed-effects models and treating participant-specific intercepts and linear change with time as random effects. This approach allowed us to assess the physical activity program (the key fixed effect) on the average rate of change in total, outpatient, and inpatient medical expenditure while accounting for the dependence of within-participant repeated measures over time. The mixed-effect models included age (continuous), sex (male and female), and intervention status (intervention or control group). Based on previous studies, all the data for medical expenditure were presented as least-squares means (95% confidence interval [95% CI]) per year during the 3-year intervention period.

We also evaluated the odds ratio (OR) and 95% CI for outpatient visits and hospitalization using a generalized linear model after adjusting for age, sex, and baseline medical expenditure. To determine changes in physical activity, physical fitness, and body composition produced by the intervention, we performed repeated measures analysis of variance (ANOVA) for the total step count and brisk walking; we conducted post hoc analysis using the Bonferroni test (baseline versus after 6, 9, and 15 months). We performed all statistical analyses with SPSS; we considered *P* < 0.05 statistically significant.

## 3. Results

At baseline, the total medical expenditure for the intervention group was $822.8, which is lower than the total medical expenditure of $2,008.9 for residents of Kazo.  Table 1 presents the results related to age, sex, proportion of elderly individuals, and total, inpatient, and outpatient medical expenditure between the intervention and control groups. We did not observe any significant difference between the two groups.

Table 2 shows the results of the mixed-effect models adjusted for age and sex. We examined the longitudinal association between medical expenditure and participation in the physical activity program. We found total and outpatient medical expenditure to be significantly higher in the control than in the intervention group. Compared with the intervention group, the control group had total medical expenditure of $640.4 (95% CI, 25.1 to 1386.3; *P* = 0.02) and outpatient medical expenditure of $361.9 (95% CI, 173.1 to 622.1; *P* = 0.002). However, we observed no significant difference for inpatient medical expenditure (*P* = 0.18).

We used generalized linear models to evaluate the OR for visiting hospital for medical care with respect to participation in the physical activity program (Table 3). We observed a significantly increased OR for outpatient visits in the control group (OR, 6.47; 95% CI, 3.55 to 11.81) compared with the intervention group; however, we did not find this for inpatients (OR, 1.16; 95% Cl, 0.49 to 2.75).

At baseline, the average total and brisk-walking steps were, respectively, 7171 (95% Cl, 6087 to 8256) and 2217 (95% Cl, 1422 to 3012) steps/day. The total step count significantly increased to 10,120 (95% Cl, 9120 to 11,121) steps/day after 6 months, 8697 (95% Cl, 7743 to 9650) steps/day after 9 months, and 8652 (95% Cl, 7795 to 9508) after 15 months (Table 4). The brisk-walking step count showed a significant increase to 3413 (95% Cl, 2553 to 4273) steps/day after 6 months, 2927 (95% Cl, 2090 to 3764) steps/day after 9 months, and 2710 (95% Cl, 2039 to 3382) after 15 months. Adherence to the physical activity program was 73.5% in the intervention group over the 15 months.  Table 5 shows the results of change in body composition and physical fitness at baseline and after 6, 9, and 15 months. We observed increased muscle mass, an increase for all items of physical fitness, and decreased fat mass and BMI (Table 5). We also performed a sensitivity analysis for the effects of change in body composition and physical fitness on change in medical expenditure for intervention program participants. However, we could not find any significant association between change in muscle mass, fat mass, and physical fitness and change in total medical expenditure (data not shown).

## 4. Discussion

This study investigated the effects of a health promotion program, which took the form of a community-based intervention for increasing physical activity, on medical expenditure. To our knowledge, this is the first investigation to use an intervention to examine the effects of a physical activity program on change in medical expenditure. Our results indicate that participation in the physical activity program was associated with decreased medical expenditure—particularly total and outpatient medical expenditure. This decreased expenditure was probably due to the reduced number of outpatient visits as a result of promoting the health status of participants.

In Japan, local governments have implemented health promotion programs to halt increasing medical expenditure; some studies have demonstrated that increased physical activity clearly decreased the risk of lifestyle diseases [13, 14, 24]. However, the health promotion programs of local governments have been conducted as single events or over very short periods; there has thus been limited opportunity for residents to participate regularly in physical activity programs [24]. Previous studies have shown that physical activity undoubtedly contributes to decreased mortality and reducing the risk of morbidity and physical disability [25]. In the present study, we observed a significant increase in physical activity and muscle mass as well as decreased BMI in the intervention group. Compared with nonobese individuals, obese people commonly have greater physical disability and higher medical expenditure [26]. Furthermore, evidence suggests that increasing physical activity is associated with a lower risk of obesity, metabolic disease, and physical disability [20, 22]. Our findings thus indicate that the health improvements achieved by increasing physical activity are possibly linked to a decrease in total and outpatient medical expenditure.

In this study, we found a significant difference in total and outpatient medical expenditure between the intervention and control groups. Compared with the control group, total medical expenditure for the intervention group was lower by $640.4 and outpatient medical expenditure was lower by $361.9. We could not directly compare our results with those of previous epidemiological investigations owing to differences in the study methods and participant characteristics; however, the reduced economic cost effect we observed in the intervention group was 44.6%, which is higher than the reduced medical costs that were 13.0% reported in one cohort study [13]. Over the 3-year observation period in the present study, the OR for hospital care was 6.47-fold higher in the control group than in the intervention group. One study for lifestyle and medical expenditure has demonstrated that participants who walked for 1 hour or more a day had lower medical expenditure than those who walked for less than that time [25]. Using a simulation study, Keeler et al. reported that the lifelong medical expenditure of individuals with a sedentary lifestyle could be reduced through exercise [27]. These previous findings are supported by the results of the present study, which used a physical activity intervention. Our findings thus suggest that a lifestyle-based health promotion program can contribute to daily active life by increasing physical activity and thereby reducing medical expenditure.

One strength of the present study is that it adopted a longitudinal design, employing a randomly sampled dataset; the analyses incorporated repeated measurements made on the same participants using mixed-effects models. However, our study also has several limitations. First, participants in the intervention group were self-sufficient, healthy, and it is possible that they were highly conscious of their health. Indeed, the participants in the intervention group had lower total medical expenditure ($882.8 annually) than the general population in Kazo ($2,008.9 annually). However, we selected our control group from among individuals of the same age and sex as well as with the same medical expenditure. Furthermore, we excluded from the present study subjects who required long-term care. We also excluded medical costs for prescription drugs and rehabilitation from total medical expenditure, even though there may have been some savings in medical costs through the physical activity program. Moreover, although the total medical expenditure for residents of Kazo included all age groups, our analysis did not cover individuals aged ≥ 75 years. However, because of the greater medical costs in older populations, it is also possible that we underestimated the effects of the physical activity program.

Another limitation of this study is that it employed a randomly selected matching control group and was not a randomized controlled study. To validate the effects of the intervention, we performed a randomly selected matching for sex, age, and place of residence. However, we did not consider other physical health conditions, healthy behavior (smoking, alcohol consumption, and dietary nutrition), or demographic factors (education levels and income).

## 5. Conclusions

In response to the social issue of increasing medical expenditure caused by Japan's aging population, it is necessary to implement health promotion programs to prevent lifestyle diseases, which account for over half of the country's total medical expenditure. We observed lower medical expenditure in participants of the health promotion program over a 3-year period than in a control group with the same medical expenditure at baseline.

## Figures and Tables

**Table 1:** Participant baseline characteristics.

	Intervention group	Control group	*P* value
	(*n* = 60)	(*n* = 300)	
Demographics			
Age (years)^†^	63.1 (4.4)	62.7 (4.5)	0.34
Sex, male (%)	36.7	41.7	0.47
Elderly (≥65 years, %)	38.3	37.7	0.92
Medical expenditures^‡^			
Total medical expenditures per capita per year ($US)	822.8 (–252.7 to 2012.4)	791.4 (167.5 to 1493.9)	0.93
Inpatient medical expenditures per capita per year ($US)	460.6 (–535.4 to 1520.6)	312.4 (197.8 to 1006.0)	0.64
Outpatient medical expenditures per capita per year ($US)	362.2 (95.2 to 679.4)	478.9 (255.5 to 597.6)	0.34

^†^Values are means (SD).

^‡^Values are least-squares means (95% CI).

Significant difference between the intervention and control groups at baseline according to *χ* ^2^ or *t*-test.

**Table 2:** Effects of physical activity intervention on change in medical expenditure over 3 years.

	Intervention group (*n* = 60)	Control group (*n* = 300)	*P* value
Total ($US/year)	0 (reference)	640.4 (25.2 to 1386.3)	0.02
Inpatient ($US/year)	0 (reference)	289.5 (–282.2 to 898.8)	0.18
Outpatient ($US/year)	0 (reference)	361.9 (173.1 to 622.1)	0.002

Mixed-effects models adjusted for age and sex.

Values are least-squares means (95% CI).

**Table 3:** Odds ratios for medical care according to participation in the physical activity program.

	Hospitalization		Outpatient visiting	
	Case/number of participants	OR (95% CI)	Case/number of participants	OR (95% CI)
Age (years, cont.)		1.03 (0.96 to 1.09)		1.03 (0.97 to 1.11)
Sex (male versus female)		0.76 (0.43 to 1.26)		0.72 (0.39 to 1.33)
Intervention status				
*Intervention group*	7/60	1.00 (reference)	28/60	1.00 (reference)
*Control group*	40/300	1.16 (0.49 to 2.75)	254/300	6.47 (3.55 to 11.81)^*∗*^

Values are odds ratio (95% CI).

^*∗*^ *P* < 0.05 versus intervention group.

**Table 4:** Time-dependent changes in total and brisk-walking step counts in the intervention group.

	Baseline	6 months	9 months	15 months	*P* value
Total steps	7171 (6087 to 8256)	10121 (9120 to 11121)^*∗*^	8697 (7743 to 9650)^*∗*^	8652 (7796 to 9508)^*∗*^	<0.001
Brisk walking	2217 (1422 to 3012)	3413 (2553 to 4273)^*∗*^	2927 (2090 to 3764)^*∗*^	2711 (2039 to 3382)^*∗*^	<0.001

Values are means (95% CI).

^*∗*^ *P* value < 0.05: repeated measures ANOVA were compared with the baseline using Bonferroni's post hoc tests. Greenhouse-Geisser correction is 0.78.

Brisk walking was defined as continuous walking for ≥10 min at a pace equivalent to ≥4 metabolic equivalents.

**Table 5:** Changes in body composition and physical fitness of participants in the intervention group.

	Baseline	6 months	9 months	15 months	*P* value
BMI (kg/m^2^)	23.2 (3.5)	22.9 (3.3)^*∗*^	22.6 (3.3)^*∗*^	22.7 (3.4)^*∗*^	<0.001
Muscle mass (%)	25.0 (3.1)	25.2 (3.2)	26.1 (3.1)^*∗*^	25.9 (3.1)^*∗*^	<0.001
Handgrip strength (kg)	27.3 (7.1)	28.5 (7.8)^*∗*^	29.6 (6.9)^*∗*^	28.9 (7.9)^*∗*^	<0.01
Sit-up (times/30 sec)	9.1 (4.9)	12.5 (6.3)^*∗*^	14.3 (5.9)^*∗*^	13.8 (6.5)^*∗*^	<0.001
Sit-and-reach (cm)	35.2 (6.8)	39.1 (7.6)^*∗*^	40.6 (8.1)^*∗*^	38.8 (7.6)^*∗*^	<0.001
One-leg standing with open eyes (sec)	98.1 (37.7)	101.2 (32.8)	111.5 (26.1)^*∗*^	108.3 (28.6)	<0.05
Walking speed (sec/10 m)	5.9 (1.0)	4.9 (1.0)^*∗*^	4.7 (0.7)^*∗*^	4.9 (0.8)^*∗*^	<0.001
Endurance test (W/kg)	1.4 (0.4)	1.5 (0.4)	1.6 (0.4)^*∗*^	1.7 (0.8)^*∗*^	<0.01

Values are means (SD).

^*∗*^ *P* value < 0.05: repeated measures ANOVA were compared with the baseline using Bonferroni's post hoc tests.
